# Characterization of Maturation-Associated Genes in Ovary–Hepatopancreas Transcriptome and *Vitellogenin* Expression in Pacific Blue Swimming Crab *Callinectes arcuatus* During Gonadal Maturity Stages

**DOI:** 10.3390/ani15192860

**Published:** 2025-09-30

**Authors:** Araceli Lorena Montes-Dominguez, Jesus Arian Avena-Soto, Martin Ignacio Borrego, Laura Rebeca Jimenez-Gutierrez

**Affiliations:** 1 Facultad de Ciencias del Mar, Universidad Autónoma de Sinaloa, Paseo Claussen s/n, Mazatlán 82000, Mexico; loremontes11@gmail.com (A.L.M.-D.); arianavena28@gmail.com (J.A.A.-S.); maribo@uas.edu.mx (M.I.B.); 2 Secretaría de Ciencias, Humanidades, Tecnología e Innovación, Ciudad de México 03940, Mexico

**Keywords:** *Callinectes arcuatus*, genic expression, gonadal maturity, protein characterization, transcriptome

## Abstract

The swimming crab is a commercially and nutritionally important marine resource with the highest catch volumes in Mexico occurring along the East Pacific coast. Among the Pacific species of the genus *Callinectes*, the blue crab *C. arcuatus* has the widest distribution and is found throughout the year. Its close resemblance to the well-studied Atlantic blue swimming crab (*C. sapidus*) makes it an excellent model for molecular reproductive studies in the Mexican Pacific. Using next-generation sequencing, this study aimed to characterize maturation-associated genes in an ovary–hepatopancreas transcriptome of *C. arcuatus*, with a particular focus on *vitellogenin* (*Vtg*) and its expression in the ovaries and hepatopancreas across different gonadal maturity stages. The transcriptome library generated from pooled samples produced 27,729 unigenes, of which, 196 (1.81%) were identified as reproduction-related genes. Notably, 33 of these genes, including the complete *Vtg* sequence, have not been previously reported in this species. *Vtg* expression was found to be tissue-specific, with levels in the hepatopancreas up to 13 orders of magnitude higher than in the ovary. In the hepatopancreas, *Vtg* expression increased exponentially from stage I to stage V of gonadal maturity, whereas in the ovaries, its expression showed the opposite trend. These findings highlight that the hepatopancreas, with its abundant nutrient reserves, serves as the primary site of *Vtg* expression and synthesis.

## Simple Summary

Swimming crabs, which belong to the genus *Callinectes*, are highly valued species and considered a delicacy worldwide, underscoring their importance to both fisheries and aquaculture. Along the Mexican Pacific coast, the blue crab *C. arcuatus* is present year-round. Despite its culinary appeal and commercial value, molecular studies on this species are limited, particularly those related to its reproduction. In this study, next-generation sequencing was used to investigate the genes and proteins involved in vitellogenesis (the synthesis and accumulation of yolk in the ovaries) in female crabs, a process closely linked to maturation. A total of 196 different reproduction-related proteins were identified, 33 of which have not been previously reported in this species, and are now available to the scientific community. For example, vitellogenin, a key protein essential for crab reproduction, was found in both the ovaries and hepatopancreas (a nutrient storage organ). Interestingly, the hepatopancreas was identified as the primary site of vitellogenin synthesis.

## 1. Introduction

The significant capture volume of crab places them as the tenth most important resource in Mexico’s fishing industry [1,2], generating USD 60 million in 2019 for an increasingly expanding sector [3]. More than half of the national production comes from the Mexican Pacific Ocean (MPO), with Sinaloa (38.18%, over 20,000 tons) and Sonora (16.65%, over 10,000 tons) being the primary contributors [2,4]. The MPO crab fishery relies on three species of the genus *Callinectes*: *C. arcuatus*, *C. bellicosus*, and *C. toxotes*. Among these, the Pacific blue crab *C. arcuatus* has the widest distribution and is present year-round [5], making it the species with the greatest aquaculture potential.

Achieving captive reproduction is essential for ensuring the sustainability of crab cultivation and, to this end, understanding female gonadal maturity is critical. Unlike males, females crabs mate only once in their lifetime and store sperm for subsequent spawning [6]. To evaluate fishery sustainability and support the implementation of seasonal closures, ecological and reproductive studies are conducted. These include egg, embryo, and larval counts, an assessment of larval viability, and evaluations of gonadal maturity stages. Several methodologies are employed for this purpose, including the gonadosomatic index, morpho-colorimetric techniques, histological analysis, and molecular markers [7]. While morpho-colorimetric and histological methods are commonly used in fisheries research, molecular approaches are more prevalent in aquaculture and require careful sample preservation.

Most molecular studies on the reproductive biology of crustaceans focus on characterizing nucleotide and amino acid sequences, as well as determining gene expression patterns related to vitellogenesis in females [7,8]. Vitellogenesis refers to the synthesis and accumulation of yolk proteins, primarily vitellins (Vn), which are essential for embryo development. Among these, vitellogenin (Vtg), the precursor of Vn, has been is extensively studied. Vtg is synthesized not only in the ovaries but also in the hepatopancreas, from where it is transported to the ovaries and accumulated to support oocyte growth and maturation [9]. The expression of the *Vtg* gene and the concentration of the Vtg protein at different gonadal maturity stages have been investigated in cultured shrimp, lobster, and crab species [7].

The Atlantic blue crab *C. sapidus* have well-established cultures, and many of their maturation-associated genes have been characterized and made publicly available in databases such as the National Center for Biotechnology Information (NCBI) [9,10]. Transcriptomic studies in cultured crustacean species have expanded our understanding of maturation-associated proteins. In addition to Vtg, proteins involved in processes such as mitosis, meiosis, hormonal regulation, and nutrient uptake have been reported [10,11]. However, the molecular mechanisms that regulate reproduction in wild crab species remain poorly understood.

Although pilot trials have been conducted for the commercial-scale cultivation of *C. arcuatus* in the Pacific Ocean, no molecular studies have been carried out on any crab species from the MPO, whether in aquaculture or fisheries. Therefore, the Pacific blue crab *C. arcuatus* was selected as a model species to initiate molecular studies in wild MPO crabs. The aim of this study was to analyze the ovary–hepatopancreas transcriptome of *C. arcuatus*, characterize its Vtg gene, and determine *Vtg* expression levels in the ovary and hepatopancreas at different stages of gonadal maturity. This work reports the number of unigenes obtained, identifies maturation-associated proteins in *C. arcuatus*, and discusses the significance of the findings.

## 2. Materials and Methods

Samples were obtained with the assistance of local fishermen, each holding a personal collection permit issued by CONAPESCA (Comisión Nacional de Pesca y Aquaculture, the National Commission of Fisheries and Aquaculture in Mexico). To capture mRNA transcription profiles over the course of an entire year, five live *C. arcuatus* females were collected monthly from Mazatlán, Sinaloa, between 2018 and 2019 (41.55 ± 11.15 g). All animals were handled in accordance with ARRIVE (Animal Research: Reporting of In Vivo Experiments) guidelines. As soon as the organisms were received, they were taken to the laboratory and euthanized by freezing and then dissected to collect hepatopancreas and ovarian tissues. Gonadal development stages were determined using morpho-colorimetric methods previously established [12] (Appendix A). The tissues were preserved at −20 °C until further use.

### 2.1. RNA Isolation, Illumina Sequencing and Bioinformatic Analyses

To obtain a broad range of reproductive transcripts, total RNA was extracted from each tissue (ovaries and hepatopancreas) of each individual, and RNA from all samples collected throughout the year was pooled for next-generation sequencing (NGS). Total RNA was extracted from 100 mg of each tissue using the PureLink RNA Mini Kit, following the manufacturer’s instructions (Thermo Fisher Scientific, Waltham, MA, USA). The RNA was processed in no more than a year. The integrity of ribosomal RNA was verified under native conditions using 1.5% agarose gels in 1 × TAE buffer with 0.1% SYBR Safe (Invitrogen, Waltham, MA, USA). Electrophoresis was performed using a Mini-Sub Cell GT System chamber (Bio-Rad, Hercules, CA, USA). Degraded RNA samples were discarded; only high-quality RNA was used for further analysis. All RNA was treated with DNase I (Roche, Basel, Switzerland; 1 U/µg RNA) to remove genomic DNA, according to the manufacturer’s instructions.

The pooled RNA sample, containing small quantities of RNA from each individual, was submitted to Genoma Mayor (Santiago de Chile, Chile). Library construction was performed using the TruSeq Stranded mRNA protocol (Illumina, San Diego, CA, USA), and sequencing was carried out on an Illumina MiSeq plataform, following the manufacturer’s instructions. The initial de novo assembly and gene ontology (GO) identification were conducted as previously described [7].

A second de novo assembly and GO identification were carried out to validate the sequences. Adapter sequences and low-quality reads were removed using Trimmomatic version 0.39. Read normalization was performed using the script <insilico_read_normalization.pl> (Trinity version 2.15.1). De novo transcriptome assembly was performed using Trinityrnaseq, and open reading frames (ORFs) were predicted using TransDecoder version 5.5.0. To establish functional annotations, BLASTx (Basic Local Alignment Search Tool version 2.17.0) alignment was conducted using Diamond version 2.1.7 against the “Nucleotide collection” databases.

GO analysis was conducted using Diamond version 0.9.22 with references from international databases, including the NCBI, UNIPROT, and the Kyoto Encyclopedia of Genes and Genomes (KEGG). Finally, sequences of interest related to reproductive function were individually verified for a third time using the BLAST algorithm available through the NCBI.

### 2.2. mRNA Expression of C. arcuatus Vtg

For gene expression analysis, at least four females sampled throughout the year were used for each maturity stage, all above the minimum catching size (120 mm) and not in an ovigerous state. Both the females and their ovaries were weighed, and the gonadosomatic index (GSI) was calculated as follows:

$$GSI=\left(\frac{Gonad\;weight}{Total\;body\;weight}\right)\times 100$$

Specific primers were designed for *β-actin*, used as a housekeeping gene, and for the triple-verified *C. arcuatus Vtg* sequence using the FastPCR program (Appendix B). Total RNA was individually extracted as described above. Complementary DNA (cDNA) was synthesized using high-quality and DNAse-treated RNA, no more than one month after being extracted, using the FirstStrand commercial kit (Invitrogen, Waltham, MA, USA) following the manufacturer’s instructions. The PCR amplification of each gene was carried out using the TopTaq PCR Master Mix kit (QiagenTM, Venlo, The Netherlands) with the following components: 12.5 μL TopTaq mix, 2.5 μL Coral dye, 1 μL of each 20 μM primer, cDNA template from each tissue, and Milli-Q water to a final volume of 25 µL. PCR products were analyzed on 1.5% agarose gels and digitized using a Gel Doc^TM^ EZ photodocumenter (Bio-Rad, Hercules, CA, USA).

Initially, *Vtg* amplification was performed, alongside the constitutive *β-actin* gene from hepatopancreas and ovarian tissues. These samples were purified using NucleoSpin columns (Macherey-Nagel, Düren, Germany), following the manufacturer’s instructions. The concentration of the purified products was quantified using a NanoDrop spectrophotometer (Thermo Fisher Scientific, Waltham, MA, USA). For absolute expression quantification, two samples with two replicates each were amplified per stage, resulting in four sub-replicates.

*Vtg* from each maturity stage was then amplified, after which the amplicons were visualized alongside the purified products to determine their absolute expression in agarose gel. Absolute expression was quantified based on the optical density of the previously purified product of known concentration as a control; this value was assigned to the product of the amplification of this gene, with the help of Image^TM^ Lab 2.0 software (Bio-Rad, Hercules, CA, USA). Relative expression was then calculated using the 2^−ΔΔCT^ method with modifications according to [9], where the *β-actin* gene served as the internal control and stage I was the reference point (time zero).

### 2.3. Statistical Analysis

The data normality and homoscedasticity of *Vtg* expression values were verified, and a one-way analysis of variance (ANOVA) was conducted with the stage of gonadal development as an independent variable. Post hoc comparisons of means were performed using the Tukey–Kramer test (*p* < 0.05). All statistical analyses were conducted using the General Linear Model module in STATISTICA (version 5.5, StatSoft, Tulsa, OK, USA).

## 3. Results

### 3.1. C. arcuatus Transcriptome

**Figure 1:**
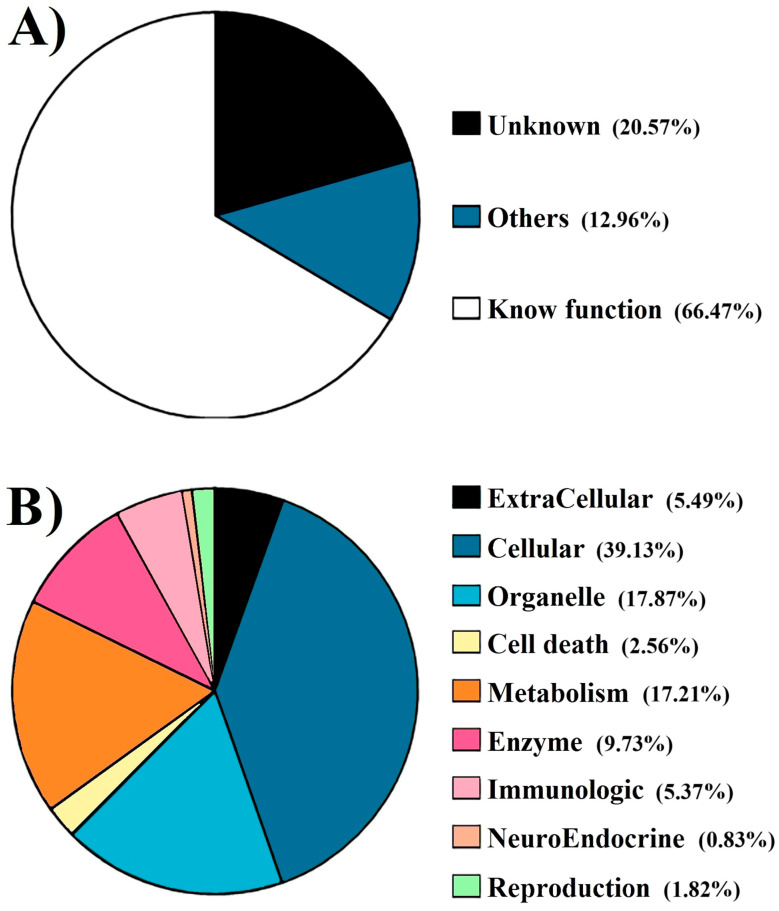
Gene ontology classification of assembled unigenes of *C. arcuatus* ovaries. (**A**) Percentage of genes with known and unknown functions. (**B**) Classification according to physiological pathways.

Female body weights range from 26.73 to 52.07 g, and ovaries weights ranged from 0.49 to 6.58 g. The combined transcriptome from ovarian and hepatopancreatic tissues comprised 27,730 unigenes. Of these, 17,272 unigenes (66.47%) were assigned known functions, 20.57% had unknown functions and the remaining unigenes contained identifiable motifs or domains but lacked specific functional annotations; these were classified as “others” (Figure 1). To improve functional classification, the unigenes with known functions were grouped into categories such as “extracellular”, “cellular”, “organelle”, “cell death”, “energy metabolism”, “enzymes”, “neuro-endocrine”, “immunologic”, and “reproduction”. The majority of unigenes were assigned to the cellular category (39.12%), followed by organelle (17.86%) and energy metabolism (17.2%). In contrast, the neuro-endocrine (0.83%) and reproduction (1.82%) categories contained the fewest unigenes. Within the reproduction category, 47.88% of unigenes were associated with molecular functions, 27.88% with biological processes, and the remainder with cellular components (Appendix C).

A total of 315 unigenes were directly or indirectly related to reproduction, encoding for 196 triple-confirmed proteins. Among these, 33 proteins have not been previously reported in *C. arcuatus*. Examples of partial protein sequences include nucleotide-binding protein G(i) subunit alpha, zinc finger homeobox protein 3, homeobox protein SIX1, NEDD8 activating enzyme E1 regulatory subunit, prostaglandin E synthase 2, Rho-associated protein kinase 2, protein singles bar, small ubiquitin-related modifier (isoforms 1 and 3), transcription factor Sox-8, spermatogenesis-defective protein 39, tolloid-like protein 2, tudor domain-containing protein 7, and zwei Ig domain protein zig-8.

Complete sequences of proteins identified for the first time in this species include brahma-associated protein of 60 kDa (brahma AP), mitogen-activated protein kinase (MAPK; isoform 4 and 14), enhancer of split m7 protein (ESm7P), germ cell-less protein, forkhead box protein (FOX; E3, K2, and N4, the last one with only a partial sequence), RNA-binding protein fox-1 homolog 3, guanine nucleotide-binding protein G (G proteins; subunit 3, (o) subunit alpha, and (q) subunit alpha), hemicentin-1, homeobox proteins (Nkx-2.2, SAX-1, MSX-2 and MSX-3), HSP90 and HSP90-alpha, maternal embryonic leucine zipper kinase (MELZK), NEDD8-conjugating enzyme, Ubc12, and several members of the prostaglandin family, including hematopoietic prostaglandin D synthase and prostaglandin reductase 1. Additional proteins identified include sex-lethal homolog (SxL), sperm-specific protein PHI-2B (SpermSP), and transcription factor Sox (TFSOX; 2 and 14).

**Figure 2:**
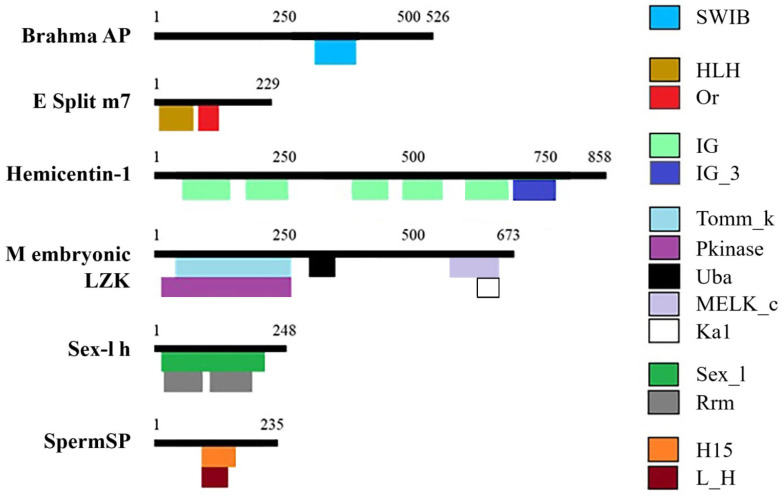
Domains present in reproduction proteins from an ovary–hepatopancreas transcriptome of *C. arcuatus*. Brahma AP: brahma-associated protein. E. split m7: enhancer of split m7 protein. M embryonic LZK: maternal embryonic leucine zipper kinase. Sex-l h: sex-lethal homolog. SpermSP: sperm-specific protein PHI-2B. SWIB: SWIB/MDM2 domain. HLH: Helix-loop-helix DNA-binding domain. Or: orange domain. IG: immunoglobulin domain. IG_3: Third immunoglobulin (Ig)-like domain of the L1 cell adhesion molecule. Tomm_k: TOMM system kinase/cyclase fusion protein. Pkinase: protein kinase domain. Uba: UBA domain. MELK_c: catalytic domain of the serine/threonine kinase. Ka1: kinase-associated domain 1. Sex_l: sex-lethal family splicing factor. Rrm: RNA recognition motif. L_H and H15: linker histone H1 and H5 family.

The signature domains of each protein were identified from individual sequences (Figure 2). The SWIB domain (involved in chromatin remodeling and transcription regulation) was detected in brahmaAP. The orange domain (a transcription repressor) was found in ESm7P. Hemicentin contained several immunoglobulin domains. MELZK featured multiple kinase domains and a UBA domain (Ubiquitin-Associated domain). The sex-lethal homolog possessed both a sex-lethal family splicing factor domain and an RNA recognition motif, while SpermSP contained a linker histone domain.

**Figure 3:**
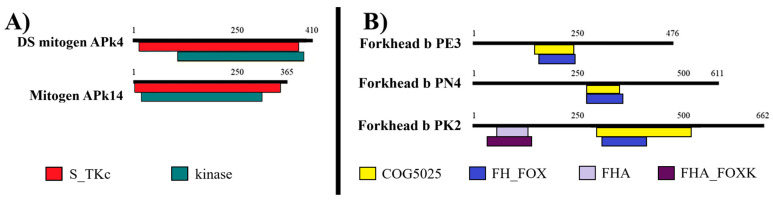
Domains present in family proteins from *C. arcuatus*: mitogen-activated protein kinase and forkhead box protein, guanine nucleotide-binding protein and homeobox. (**A**) Mitogen-activated protein kinase family. (**B**) Forkhead box protein family. DS mitogen-AP kin. 4: dual specificity mitogen-activated protein kinase 4. Mitogen-AP kin. 14: mitogen-activated protein kinase 14. S_TKc: serine/threonine protein kinases. Forkhead b P: Forkhead box protein (members E3, N4 and K2). COG5025: transcription factor of the Forkhead/HNF3 family. FH_FOX: forkhead (FH) domain. FHA: forkhead-associated (FHA) domain.

Among proteins families, both MAPK isoforms shared the serine/threonine protein kinase domain. FOX proteins contained the forkhead associated domain and COG5025 domain (transcription factor; Figure 3).

**Figure 4:**
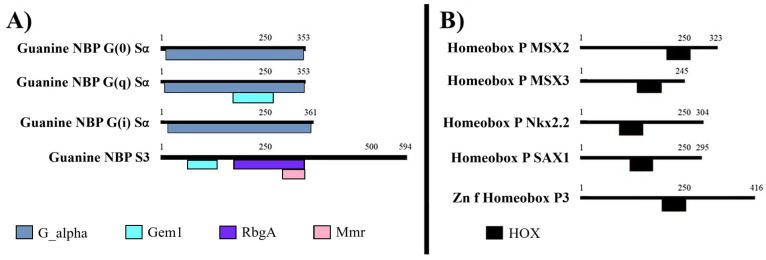
Domains present in family proteins from *C. arcuatus*: guanine nucleotide-binding protein and homeobox. (**A**) Guanine nucleotide-binding protein family. (**B**) Homeobox protein family. Guanine NBP: guanine nucleotide-binding protein (member S3 and members of Sα G(0), G(q), G(i)). Homeobox P: homeobox protein (members MSX2, MSX3, Nkx2.2, SAX1 and zinc finger homeobox protein 3). G_alpha: G-protein alpha subunit. Gem1: GTPase SAR1 family domain. RbgA: ribosome biogenesis GTPase RbgA. Mmr: 50S ribosome-binding GTPase. HOX: Homeodomain.

GNBP isoforms shared the G-protein alpha subunit domain; however, only GNBP3 also possessed the RbgA (Ribosome biogenesis GTPase RbgA) and MMR (50S ribosome-binding GTPase) domains. Homeobox isoforms featured only have the HOX domain (DNA binding; Figure 4).

**Figure 5:**
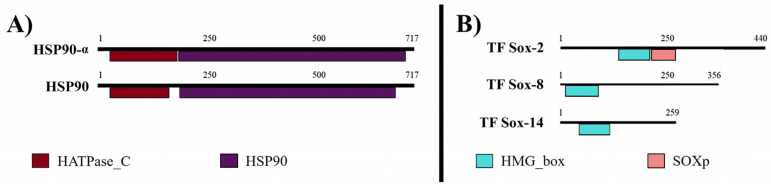
Domains present in family proteins from *C. arcuatus*: HSP90 and Transcription factor Sox. (**A**) Heat shock protein 90. (**B**) Transcription factor Sox. HSP90: heat shock protein 90. HSP90-α: heat shock protein 90 subunit α. TF Sox: transcription factor Sox (members 2, 8 and 14). HATPase_C: histidine kinase-like ATPases. HSP90: histidine kinase-like ATPase domain. HMG_box: high-mobility group (HMG)-box. SOXp: SOX transcription factor.

**Figure 6:**
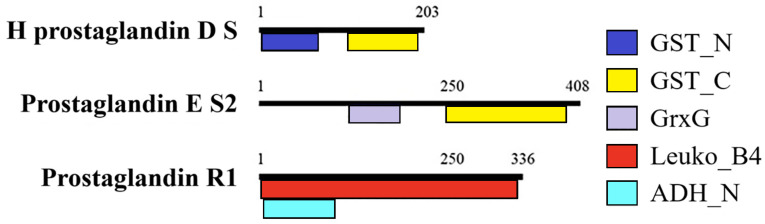
Domains present in family prostaglandin biosynthesis proteins from *C. arcuatus*. H prostaglandin D S: hematopoietic prostaglandin D synthase. Prostaglandin E S2: prostaglandin D synthase. Prostaglandin R1: prostaglandin reductase. GST_N: Glutathione S-transferase, N-terminal domain. GST_C: Glutathione S-transferase, C-terminal domain. GrxG: Glutaredoxin. Leuko_B4: 15-prostaglandin reductase and leukotriene B4 12 hydroxydehydrogenase activity. ADH_N: N-terminal domain of oxidoreductase.

Additionally, isoforms of HSP90 and TFSOX exhibited domains characteristic of their respective protein families (Figure 5), whereas the prostaglandin biosynthesis-related proteins were not isoforms and shared few, if any, conserved domains (Figure 6).

**Figure 7:**
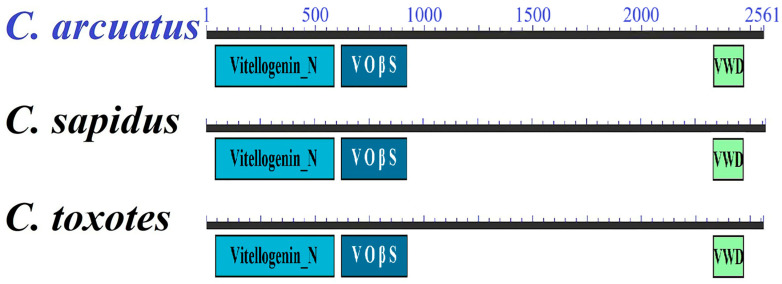
Domains present in the Vtg protein of crabs of the genus *Callinectes*. *C. arcuatus* (QGA73525.2), *C. sapidus* (AEI59132.1) and *C. toxotes* (QGA73526.1). VOβS: Vitellinogen, open beta-sheet. VWD: von Willebrand factor.

Additionally, previously reported partial sequences of the clathrin heavy chain 1 and nuclear progesterone receptor were extended in this study, although both sequences remain incomplete. In contrast, the previously partial sequence of *Vtg* was successfully completed and has been updated in the GenBank database (MN105880.2). The same Vtg sequence was identified in both the ovary and hepatopancreas of *C. arcuatus*. The complete deduced Vtg sequence consists of 2561 amino acid residues. It contains the Vitellogenin_N domain spanning residues 41 to 586, the Vit_open_b-sht domain (VOβS) from residues 619 to 920, and the VWD domain from residues 2329 to 2469. Additionally, the RERR endoprotease cleavage site is located within the VoβS domain, specifically between residues 706 and 709 (Figure 7).

### 3.2. mRNA Expression of C. arcuatus Vtg

The GSI ranged from 0.93% to 11.14% across gonadal maturity stages I to V, respectively (Table 1). The relative expression of *C. arcuatus Vtg* showed significant variation across stages. In the ovaries, *Vtg* expression exhibited a decreasing trend, with an average value of 1.005 at stage I, gradually declining to nearly undetectable levels by stage V (Table 1). Statistically significant differences were observed among all maturity stages. Overall, ovarian *Vtg* expression was lower at every stage when compared to expression in the hepatopancreas.

In the hepatopancreas, *Vtg* expression values were consistently higher and showed an opposite trend to those observed in the ovaries. The lowest expression occurred at stage I, with an average value of 1.15, followed by an exponential increase through to stage V, where expression levels reached up to 13 orders of magnitude higher than those at stage I.

## 4. Discussion

The commercial and nutritional value of crabs is widely recognized, highlighting the importance of ensuring their sustainability for future generations [4]. Evaluating reproductive parameters is essential for both fisheries and aquaculture management. In fisheries, such assessments enable the determination of minimum catch sizes, maturation and spawning periods, and the implementation of closed seasons to help maintain adequate breeding populations [4,13]. In aquaculture, increasing attention has been given to the evaluation of spawning induction methods, genetic improvement programs, and molecular tools.

Similarly to its Atlantic counterpart *C. sapidus*, the Pacific blue crab *C. arcuatus* shows considerable potential for cultivation. To expand our understanding and effective use of this species, establishing a solid foundation for evaluating its biological and reproductive characteristics is crucial in both fisheries and aquaculture contexts. Like other *Callinectes* species, *C. arcuatus* displays seasonal variations in behavior, growth, and reproduction, typically intensifying during warmer months. However, these patterns are often species-specific [14]. Notably, females invest more heavily in reproduction than males, undergoing a prolonged and energetically demanding physiological process [11,14].

Sexual reproduction in crabs is a finely regulated process that extends beyond the biosynthesis of proteins in the gonads. NGS has significantly accelerated the identification of genes involved in reproductive processes compared to traditional Sanger sequencing: 1. Directly reproduction-related proteins include those involved in vitellogenesis, gonadal maturation, meiosis, oocyte regulation, sex differentiation, and embryonic development. 2. Indirectly reproduction-related proteins, such as neurotransmitters, neuropeptides, and hormone receptors, and nutrient regulators, play critical roles through autocrine and/or paracrine signaling mechanisms [11].

Previously, our lab group uploaded to the NCBI database more than 100 genes from *C. arcuatus* with a possible relationship with reproduction, including *Vtg receptor* (MW685989.1), *fem-1 C* (MT488353.1), *VASA* (MT488368.1), mab-21 (MT488382.1), *slowmo* (MT488367.1), *ovarian lipoprotein receptor* (MT488366.1), *nuclear progesterone receptor* (MT488349.1), and *vitelline envelope zona pellucida* (MT488345.1), among many others. For most of them, their function and relation with reproduction have been summarized by [9].

More than 30 previously unreported genes were identified in *C. arcuatus*, several of which are involved in key processes related to reproduction and embryonic development. MELKZ is a critical regulator of embryogenesis, implicated in asymmetric cell division [15]. MAPKs are conserved serine/threonine protein kinases that promote meiotic activation and oogonia differentiation [16,17]. Proteins involved in the prostaglandin pathway contribute to various reproductive functions and regulate the process of mitosis and meiosis during ovarian development, along with the release of hatching factors and ovarian maturation in crustaceans [8,18]. Both Sxl and TFSOX are involved in sexual differentiation [19,20], making them particularly relevant given the growing interest in monosex culture practices, which are especially valuable in crab aquaculture. Additionally, Sxl also plays a role in female gonadal development [19], while TFSOX contributes to embryonic development [20].

Hemicentin is secreted by skeletal muscles and gonad leader cells and has been proposed to be essential for the stabilization of the germline syncytium. In zebrafish embryos, it promotes the attachment of the epidermal and somite basement membranes to the extracellular matrix. As we found herein, hemicentin has a long chain of immunoglobulin modules [21]. Brahma AP is a component of the complex SWItch/Sucrose Non-Fermentable; various members of this protein family are involved in essential cellular processes such as transcriptional activation, DNA repair and recombination, and transcriptional repression. Additionally, it plays a role in heart development [22]. In contrast, the enhancer of split is often regulated by Notch cell signaling and is important for neurogenesis [23].

Some of the genes identified in this study are indirectly associated with the regulation of reproduction and vitellogenesis. Zinc finger homeobox (Zfhx1/Z81) is one of the earliest transcription factors expressed during the gastrula stage in serotonergic neural precursor cells and is also required for serotonin synthesis [24]. FOX genes regulate cholesterol metabolism and the steroid hormone pathway, both essential for nutrient uptake during vitellogenesis [17]. G proteins function as signal transducers for various hormones and neurotransmitters, thereby influencing systemic processes such as embryonic and gonadal development [25]. Hsp90 contains a conserved histidine kinase-like ATPase domain; this protein enhances the activity of the estrogen receptor complex, promoting the transcription of target genes, including those involved in nutrient uptake and Vtg synthesis, one of the most critical proteins in gonadal development [26]. Together, these genes act in a coordinated and finely regulated manner to control various aspects of reproduction, including follicle cell development, oogonia differentiation, oocyte proliferation, vitellogenesis, and ovarian maturation [17]. Based on these findings, they may serve as valuable targets for future studies aimed at achieving the successful captive reproduction of *C. arcuatus*, particularly through the analysis of their expression patterns in the ovary and hepatopancreas.

Since the early 2000s, extraovarian Vtg synthesis has been widely reported in various species [27]. In most crustaceans, the hepatopancreas is considered the primary site of extraovarian Vtg synthesis [28]. This is consistent with the findings of the present study, as GSI values correspond more closely with *Vtg* expression levels in the hepatopancreas than in the ovary. Canonically, extraovarian Vtg is secreted into the hemolymph and taken up by the ovaries via endocytosis [29]. However, a recent study in *Penaeus vannamei* suggests that the anatomical proximity between the ovaries and hepatopancreas, along with the presence of Vtg receptors (RVtg) on the membranes of both organs, may indicate a more direct route of communication [9].

Multiple Vtg isoforms have been reported in some species, though this must be evaluated on a species-by-species basis. In cases where two isoforms have been identified, they are often immunologically indistinguishable [14]. The nucleotide sequence length of *Vtg* in various crustacean species typically ranges from 5000 to 7000 nucleotides [7]. A phylogenetic tree constructed using Vtg sequences previously deposited in GenBank from different crustacean species revealed no clear separation by isoform [7]. Rather, potential isoforms from the same species clustered within the same node. The domains within each Vtg protein are highly conserved in both location and length, with sequence identities among crab species ranging from 89% to 97%. This suggests that while isoforms may exist, they likely share a common ancestor, and any differences are probably due to point mutations [30].

In this study, we obtained the complete *Vtg* sequence for *C. arcuatus*. This sequence matches the length of the *Vtg* found in the Pacific black crab *C. toxotes* (MN105881.1), coding for a protein of 2560 amino acid. Among the domains in crustacean Vtg, the Vitellogenin_N domain is particularly noteworthy as it is a highly conserved lipoprotein region critical for nutrient transport and uptake and is considered the most functionally significant domain [31,32,33].

*Vtg* transcripts serve as an indirect indicator of Vtg proteins and, by extension, the degree of gonadal maturity. Gonadal maturity in crustaceans is typically categorized into four or five stages depending on the classification system used [34,35,36,37]. In this study, due to seasonal variations in reproductive patterns, not all five stages of maturity were observed in every month. Instead, more advanced stages were predominantly found during periods of higher temperatures [14]. Accordingly, it is recommended that future studies include seasonal assessments to better understand reproductive patterns in *C. arcuatus*.

Vtg synthesis, both ovarian and extraovarian, has been confirmed in numerous species, and its contribution to gonadal maturation varies depending on the species [14]. In some caridean shrimp, such as *Macrobrachium rosenbergii*, *Cherax quadricarinatus*, and *Charybdis feriatus*, *Vtg* expression is restricted to the hepatopancreas [3]. In contrast, species such as the crabs *C. sapidus*, *Eriocheir sinensis*, and *S. paramamosain*, the shrimps *P. monodon* and *P. japonicus*, and the lobster *Homarus americanus* exhibit *Vtg* expression in both the ovary and the hepatopancreas [10,14,29,38].

*Vtg* expression patterns are both species-specific and tissue-specific [29]. In most crustaceans, the highest levels of ovarian *Vtg* expression occur during the early stages of gonadal maturation. However, consistent with the results of this study, expression levels are generally higher in the hepatopancreas across all maturity stages, with a tendency to increase as maturation progresses [7,10,31,33].

Moreover, *Vtg* expression is known to be influenced by a range of physiological factors, including hormonal regulation, neurotransmitters, and steroids [10,33,39], and environmental factors such as geographic location, diet, and physicochemical parameters [9]. Importantly, Vtg is not the only protein involved in gonadal maturation. Among the thousands of genes expressed in ovaries under specific physiological conditions, the majority are related to general cellular processes (e.g., mitosis, replication, transcription, and translation), while only about 2% are associated with reproduction [40], an observation consistent with the findings of this study. A wide variety of proteins are either directly or indirectly involved in reproductive processes.

Transcriptomic analyses have been instrumental in identifying many of these proteins with potential roles in reproduction [11,40]. In *P. vannamei*, for example, key reproductive-related proteins such as Vtg, RVtg, VASA, the progesterone receptor, and Fem-1 have been reported in both the ovary and hepatopancreas [9].

Most reproduction-related proteins are highly conserved and are found across a wide range of metazoans, whether aquatic, aerial, or terrestrial. Their characterization has revealed shared domains and similar sequence lengths, regardless of reproductive strategy (e.g., hermaphroditic, dioecious, internal or external fertilization, parthenogenetic). A prominent example is Vtg, which is present in organisms ranging from sponges to mammals [11].

Certain proteins involved in reproductive regulation exhibit functional relationships, either by stimulating one another or participating in reaction cascades. Additionally, many of these proteins are multifunctional [7,9,11,40]. For example, Vtg has been implicated not only in vitellogenesis but also in sexual differentiation and it has even been detected at low levels in male testes [29,33]. These findings highlight the importance of exploring the full range of functions of reproductive proteins in Pacific crabs species, including the genes that encode them. Such investigations could enhance the species’ potential for domestication and selective breeding. Importantly, these evaluations should not be limited to controlled aquaculture environments but should also consider the dynamic and complex conditions of East Pacific marine ecosystems.

## 5. Conclusions

In the transcriptome of *C. arcuatus*, maturation-associated genes account for 1.82% of the total, corresponding to 196 proteins. Among these, 33 proteins have not been previously reported in *C. arcuatus*.

The complete *Vtg* sequence of *C. arcuatus* was identified in both the ovaries and hepatopancreas. Under the evaluated conditions, *Vtg* expression in the hepatopancreas was approximately 13 orders of magnitude higher than in the ovaries. In the hepatopancreas, *Vtg* expression was lowest at stage I and increased exponentially during the later vitellogenic stages. In contrast, the expression pattern in the ovaries showed an inverse trend.

## Figures and Tables

**Table 1:** GSI and *Vtg* expression in *C. arcuatus* ovaries and hepatopancreas.

Maturity			*Vtg* Relative Expression			
Stage	GSI (%)		Ovaries		Hepatopancreas	
I	0.933	±0.38 a	1.006	±0.133 a	1.153	±0.707 a
II	3.074	±0.17 b	0.000	±0.000 b	4.2 × 10^7^	±8.2 × 10^6^ b
III	5.934	±0.51 c	0.060	±0.006 b	1.8 × 10^8^	±7.3 × 10^7^ b
IV	8.201	±0.31 d	0.010	±0.002 b	1.03 × 10^3^	±318 × 18 c
V	11.142	±1.02 e	0.000	±0.000 b	6.5 × 10^13^	±1.5 × 10^12^ d

GSI: gonadosomatic index. *Vtg* expression was analyzed using the 2^−ΔΔCT^ method with the constitutive *β-actin* gene as a reference. Values are presented as mean ± standard deviation (*n* = 4). Hp: hepatopancreas. Different letters represent significant differences between maturity stages (*p* < 0.05).

**Figure A1:**
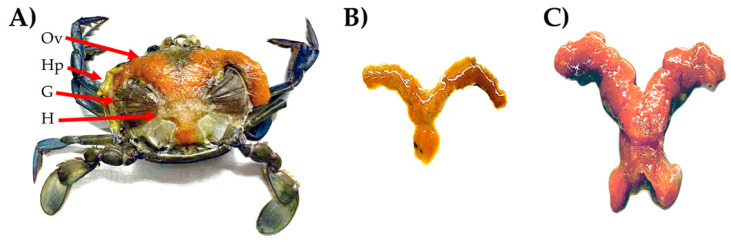
Internal organs from *C. arcuatus*. (**A**) Internal organs. (**B**) Immature ovaries (0.99 g). (**C**) Mature ovaries (6.08 g). Ov: ovaries. Hp: hepatopancreas. G: gills. H: heart.

**Figure A2:**
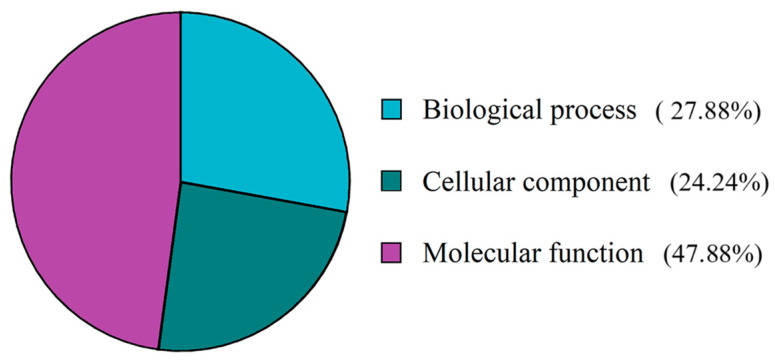
GO analysis from reproduction-related genes from *C. arcuatus*.

## Data Availability

Raw data and supplemental information for this article can be found online in Figshare at https://figshare.com/ item 10.6084/m9.figshare.23535093 (accessed on 29 August 2025).

## Appendix B

**Table A1:** Primers for *β-actin* and *Vtg* amplification.

Gen	Sequence	PCR Product (bp)	Reference
*β-actin*	Fw > AAAATGTGTGACGACGAGG	987	DQ084066.1
	Rv > GATCTTCATGGTGGAGGG		
*Vtg*	Fw > TGTCCTTCACCACAGGC	800	MN105880.2
	Rv > CAGAGCTGGAGCAAACC		

Bp: base pair.

## References

[1] CONAPESCA (2023). Anuario Estadístico de Acuacultura y Pesca 2023.

[2] SonPlayas Inician Capturas de Jaiba en Sinaloa y Sonora; Levantan Veda Anticipada. https://sonplayas.com/pesca/inician-capturas-de-jaiba-en-sinaloa-y-sonora-levantan-veda-de-forma-anticipada/.

[3] CONAPESCA Captura de Jaiba, Opción Nutritiva Ante la Veda del Camarón. https://www.gob.mx/conapesca/articulos/captura-de-jaiba-opcion-nutritiva-ante-la-veda-del-camaron-267557.

[4] INAPESCA (2014). Acuerdo por el que se da a Conocer el Plan de Manejo Pesquero de Jaiba (Callinectes spp.) de Sinaloa y Sonora.

[5] Rivera-Velázquez P.J., Aragón-Noriega E.A., Rodríguez-Domínguez G., Pérez-González R., Castillo-Vargasmachuca S.G. (2018). Growth, maturity and mortality of the blue crab *Callinectes arcuatus* Ordway, 1863 (Decapoda, Portunidae) in a Mexican coastal lagoon. Crustaceana.

[6] Diarte-Plata G. (2016). Aspectos poblacionales de las jaibas del género *Callinectes* (Decápoda: Portunidae) en la laguna El Colorado, Ahome, Sinaloa, México. College Thesis.

[7] Jimenez-Gutierrez S., Cadena-Caballero C.E., Barrios-Hernandez C., Perez-Gonzalez R., Martinez-Perez F., Jimenez-Gutierrez L.R. (2019). Crustacean vitellogenin: A systematic and experimental analysis of their genes, genomes, mRNA and proteins; and perspective to next generation sequencing. Crustaceana.

[8] Feng Q.M., Liu M.M., Cheng Y.X., Wu X.G. (2021). Comparative proteomics elucidates the dynamics of ovarian development in the Chinese mitten crab *Eriocheir sinensis*. Comp. Biochem. Physiol. D Genom. Proteom..

[9] Montes-Dominguez A.L., Avena-Soto J.A., Lizarraga-Rodriguez J.L., Perez-Gala R.J., Jimenez-Gutierrez S., Sotelo-Falomir J.A., Pinzon-Miranda F.M., Martinez-Perez F., Muñoz-Rubi H.A., Chavez-Herrera D. (2021). Comparison between cultured and wild Pacific white shrimp (*Penaeus vannamei*) vitellogenesis: Next-generation sequencing and relative expression of genes directly and indirectly related to reproduction. PeerJ.

[10] Zmora N., Trant J., Chang S., Chung J. (2007). Vitellogenin and its messenger RNA during ovarian development in the female blue crab, *Callinectes sapidus*: Gene expression, synthesis, transport, and cleavage. Biol. Reprod..

[11] Jimenez-Gutierrez L.R. (2022). Female reproduction-specific proteins, origins in marine species, and their evolution in the animal kingdom. J. Bioinform. Comput. Biol..

[12] Castañeda-Fernández de Lara V., Gómez-Rojo C., Castro-Salgado J., García-Borbón J. (2015). Buenas prácticas de pesca de jaiba guerrera *Callinectes bellicosus* en Baja California Sur, México. Cienc. Pesq..

[13] Ramírez-Félix E., Singh-Cabanillas J., Gil H., Sarmiento S., Salazar I., Montemayor G., García J., Rodríguez G., Castañeda N. (2003). La Pesquería de Jaiba (Callinectes spp.) en el Pacífico Mexicano: Diagnóstico y Propuesta de Regulación.

[14] Thongda W., Chung J.S., Tsutsui N., Zmora N., Katenta A. (2015). Seasonal variations in reproductive activity of the blue crab, *Callinectes sapidus*: Vitellogenin expression and levels of vitellogenin in the hemolymph during ovarian development. Comp. Biochem. Physiol. A Mol. Integr. Physiol..

[15] Mishra N., Wei H., Conradt B. (2018). *Caenorhabditis elegans* ced-3 caspase is required for asymmetric divisions that generate cells programmed to die. Genetics.

[16] Yu Z., Geng Y., Huang A., Wang K., Huang X., Chen D., Ou Y., Wang J. (2017). Molecular characterization of a p38 mitogen-activated protein kinase gene from *Scylla paramamosain* and its expression profiles during pathogenic challenge. J. Invertebr. Pathol..

[17] Benzhen L., Shucheng S., Chenchang B., Zhaoxia C., Yanan Y. (2024). Transcriptome analysis elucidates mating affects the expression of intra-/extra-ovarian factors, thereby influencing ovarian development in the mud crab *Scylla paramamosain*. Comp. Biochem. Physiol. D Genom. Proteom..

[18] Hansen K., Varvas K., Järving I., Samel N. (2014). Novel membrane-associated prostaglandin E synthase-2 from crustacean arthropods. Comp. Biochem. Physiol. B Biochem. Mol. Biol..

[19] Zheng J., Cheng S., Jia Y., Gu Z., Li F., Chi M., Liu S., Jiang W. (2019). Molecular identification and expression profiles of four splice variants of Sex-lethal gene in *Cherax quadricarinatus*. Comp. Biochem. Physiol. B Biochem. Mol. Biol..

[20] Yao C., Wan H., Zhang Z., Lin J., Wang Y. (2020). Genome-wide identification and expression profile of the sox gene family in different tissues and during embryogenesis in the Pacific white shrimp (*Litopenaeus vannamei*). Gene.

[21] Lan H., Wang X., Jiang L., Wu J., Wan X., Zeng L., Zhang D., Lin Y., Hou C., Wu S. (2019). An extracellular matrix protein promotes anillin-dependent processes in the *Caenorhabditis elegans* germline. Life Sci. Alliance.

[22] Akerberg B.N., Pu W.T. (2020). Genetic and Epigenetic Control of Heart Development. Cold Spring Harb. Perspect. Biol..

[23] Dearden P.K. (2015). Origin and evolution of the enhancer of split complex. BMC Genom..

[24] Yaguchi J., Angerer L.M., Inaba K., Yaguchi S. (2012). Zinc finger homeobox is required for the differentiation of serotonergic neurons in the sea urchin embryo. Dev. Biol..

[25] Neves S.R., Ram P.T., Iyengar R. (2002). G protein pathways. Science.

[26] Nguyen T.V., Ryan L.W., Nocillado J., Le Groumellec M., Elizur A., Ventura T. (2020). Transcriptomic changes across vitellogenesis in the black tiger prawn (*Penaeus monodon*), neuropeptides and G protein-coupled receptors repertoire curation. Gen. Comp. Endocrinol..

[27] Jayasankar V., Tsutsui N., Jasmani S., Saido-Sakanaka H., Yang W.J., Okuno A., Hien T.T.T., Aida K., Wilder M.N. (2002). Dynamics of vitellogenin mRNA expression and changes in hemolymph vitellogenin levels during ovarian maturation in the giant freshwater prawn *Macrobrachium rosenbergii*. J. Exp. Zool..

[28] Auttarat J., Phiriyangkul P., Utarabhand P. (2006). Characterization of vitellin from the ovaries of the banana shrimp *Litopenaeus merguiensis*. Comp. Biochem. Physiol. B Biochem. Mol. Biol..

[29] Jia X., Chen Y., Zou Z., Lin P., Wang Y., Zhang Z. (2013). Characterization and expression profile of Vitellogenin gene from *Scylla paramamosain*. Gene.

[30] Kung S.Y., Chan S.M., Hui J.H.L., Tsang W.S., Mak A., He J.G. (2004). Vitellogenesis in the Sand Shrimp, *Metapenaeus ensis*: The Contribution from the Hepatopancreas-Specific Vitellogenin Gene (MeVg2). Biol. Reprod..

[31] Ferré L.E., Medesani D.A., García C.F., Grodzielski M., Rodríguez E.M. (2012). Vitellogenin levels in hemolymph, ovary and hepatopancreas of the freshwater crayfish *Cherax quadricarinatus* (Decapoda: Parastacidae) during the reproductive cycle. Rev. Biol. Trop..

[32] Bembe S., Zmora N., Williams E., Place A., Liang D., Chung J. (2018). Effects of temperature and photoperiod on hemolymph vitellogenin levels during spawning events of the blue crab, *Callinectes sapidus*, in captivity. Aquac. Res..

[33] Bai H., Qiao H., Li F., Fu H., Sun S., Zhang W., Jin S., Gong Y., Jiang S., Xiong Y. (2015). Molecular characterization and developmental expression of vitellogenin in the oriental river prawn *Macrobrachium nipponense* and the effects of RNA interference and eyestalk ablation on ovarian maturation. Gene.

[34] Phiriyangkul P., Puengyam P., Jakobsen I.B., Utarabhand P. (2007). Dynamics of vitellogenin mRNA expression during vitellogenesis in the banana shrimp *Penaeus* (*Fenneropenaeus*) *merguiensis* using real-time PCR. Mol. Reprod. Dev..

[35] Perdichizzi A., Pirrera L., Micale V., Muglia U., Rinelli P. (2012). A Histological study of ovarian development in the giant red shrimp *Aristaeomorpha foliacea* (Crustacea: Decapoda: Aristeidae) from the Southern Tyrrhenian Sea (Western Mediterranean). Sci. World J..

[36] Pérez-Ferro D.G., Paramo-Granados J.E. (2014). Maturity stages of pink shrimp *Farfantepenaeus notialis* (Penaeidae) in the colombian Caribbean. Acta Biol. Colomb..

[37] Nguyen T.V., Rotllant G.E., Cummins S.F., Elizur A., Tomer T. (2018). Insights into sexual maturation and reproduction in the Norway lobster (*Nephrops norvegicus*) via in silico prediction and characterization of neuropeptides and G protein-coupled receptors. Front. Endocrinol..

[38] Mohd-Shamsudin M.I., Kang Y., Lili Z., Tan T.T., Kwong Q.B., Liu H., Zhang G., Othman R.Y., Bhassu S. (2013). In-depth transcriptomic analysis on giant freshwater prawns. PLoS ONE.

[39] Avena-Soto J.A., Montes-Dominguez A.L. (2022). Expresión de la *Vtg*, *RPro*, *PDO* y *FaMet* en los Estadios Gonadales del Camarón Blanco *Penaeus vannamei* (Boone, 1931) en Medio Silvestre y Cultivo. College Thesis.

[40] Gao J., Wang X., Zou Z., Jia X., Wang Y., Zhang Z. (2014). Transcriptome analysis of the differences in gene expression between testis and ovary in green mud crab (*Scylla paramamosain*). BMC Genom..

